# Progressing of a power model for electrical conductivity of graphene-based composites

**DOI:** 10.1038/s41598-023-28232-9

**Published:** 2023-01-28

**Authors:** Yasser Zare, Kyong Yop Rhee, Soo-Jin Park

**Affiliations:** 1 Biomaterials and Tissue Engineering Research Group, Department of Interdisciplinary Technologies, Breast Cancer Research Center, Motamed Cancer Institute, ACECR, Tehran, Iran; 2 Department of Mechanical Engineering (BK21 Four), College of Engineering, Kyung Hee University, Yongin, Republic of Korea; 3 Department of Chemistry, Inha University, Incheon, 22212 Republic of Korea

**Keywords:** Engineering, Materials science

## Abstract

This work presents a power equation for the conductivity of graphene-based polymer composites by the tunneling length, interphase deepness and filler size. The impressions of these factors on the effective concentration and percolation beginning of graphene nano-sheets in nanocomposites are also expressed. The developed equations for percolation beginning and conductivity are examined by the experimented data of some examples, which can guesstimate the interphase depth, tunneling size and percolation exponent. Besides, the impacts of numerous factors on the percolation beginning and conductivity are designed. The developed equation for percolation beginning shows the formation of thick interphase and large tunnels in the reported samples. So, disregarding of tunneling and interphase spaces in polymer graphene nanocomposites overpredicts the percolation beginning. Additionally, the developed model presents the acceptable calculations for the conductivity of samples. Among the mentioned parameters, the concentration and graphene conductivity in addition to the interphase depth induce the strongest effects on the conductivity of composites.

## Introduction

Many types of nanoparticles were reported in literature^1–16^. Graphene as one-atom planar sheet with nanoscale and remarkable aspects such as high electrical conductivity, significant stiffness and large specific surface area can replace the common fillers for fabrication of polymer nanocomposites^17–28^. Single graphene nano-sheets show the best intrinsic properties, but, it is problematic to gain them at high-quality, large-scale and by low-cost. Also, the tendency of graphene to rolling, scrolling or wrinkling is an important challenge, which deteriorates the aspect ratio (ratio of diameter to thickness) and morphology of graphene^29,30^.

The thin and large layers of graphene produce the conductive nets in polymer nanocomposites at low filler contents^31,32^. It is known that above a determinate filler amount in nanocomposites as percolation beginning, the nets are formed and a significant conductivity is obtained. The percolation beginning links to the dimensions of graphene layers in addition to the dispersion quality^33^. The low percolation beginning and high conductivity of polymer graphene nanocomposites are qualified by the big aspect ratio, big specific superficial zone and homogenous spreading of graphene layers^34^, although some undesirable phenomena such as aggregation, crimping and difficult networking of graphene weaken their efficiency for conductivity^35^.

There are many experimental studies in literature on the conductivity of graphene-filled samples^36–38^. They attempted to show a poor percolation beginning and great conductivity by little filler contents in different nanocomposites. However, the effects of different factors on the percolation beginning and conductivity of graphene systems were not studied. The previous articles mostly applied the power-law percolation theory to approximate the percolation beginning and to interpret the conductivity^36,38,39^. In fact, the former studies only focused on the percolation beginning in these nanocomposites, while the main effects of some important factors like interphase parts on the conductivity were neglected.

**Figure 1:**
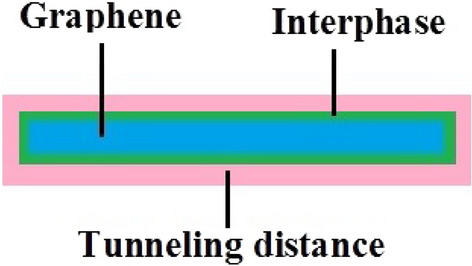
Schematic representation of interphase and tunneling regions in a sample.

Polymer nanocomposites include a third phase around nanoparticles as interphase regions^40–46^. The interphase includes the altered configuration of polymer chains near the nanoparticles, because the large surface area of nanoparticles as well as the strong interactions between polymer and nanofiller mainly affects the polymer chains near the nanofiller. So, the interphase has a higher stiffness and conductivity compared to bulk polymer chains. Figure 1 shows the interphase around the graphene in a nanocomposite. The stiffening role of interphase was discussed in the earlier studies^47,48^. Additionally, many models have been developed to calculate the interphase properties by tensile modulus and strength^49–51^. Importantly, it was shown that the interphase part contiguous nanoparticles can participate in the filler nets quickening the percolation beginning in the samples, because the interphase reduces the spaces between two nearby nanoparticles^52,53^. The networking efficiency of interphase was also studied in the toughness of CNTproducts^54^, but its effect on the conductivity has not been reported. Generally, there is no model, which can show the impact of interphase on the conductivity of graphene products. Moreover, the tunneling effect acts a chief role in the conductivity of nanocomposites^55–57^, but this mechanism cannot be considered by conventional theories. Figure 1 shows the tunneling space around nanoparticles by a schematic. The simple power-law model foresees the conductivity by filler conduction, filler sum, percolation beginning and an unclear exponent. However, the conductivity depends on many parameters such as filler shape, particle size, interphase regions, tunneling effect, which cannot be taken into account by this model.

In this work, a developed form of the power-law model is presented for approximation of conductivity in graphene systems assuming interphase depth, tunneling size and particle size. The stimuli of these factors on the percolation beginning of graphene are also expressed by a developed equation. The developed equations for percolation beginning and conductivity are evaluated by the experimented data for some examples from previous reports. Moreover, the impressions of different factors on the percolation beginning and conductivity are explored using the developed equations.

## Theoretical views

A power model based on percolation idea was advised for the conductivity of composites^36^ as:

1 $$\sigma = \sigma_{f} \left( {\varphi_{f} - \varphi_{p} } \right)^{b}$$

where “σ_f_” is filler conduction, “$\varphi_{f}$” is filler volume portion, “$\varphi_{p}$” is volume portion at percolation beginning and “b” is an exponent. This model shows a satisfactory arrangement with the tested conductivity of many nanocomposites^38,58^. The values of “b” were reported as 1.6–2 for a three-dimensional (3D) system and 1–1.3 for 2D one^58^, but more “b” range was also suggested for polymer graphene nanocomposites.

The interphase regions commonly form in polymer nanocomposites, which grow the efficiency of nanoparticles. The volume portion of interphase in the system comprising nano-sheets^59^ is obtained by:

2 $$\varphi_{i} = \varphi_{f} \left( {\frac{{2t_{i} }}{t}} \right)$$

where “t” and “t_i_” show the thickness of nano-sheets and interphase, in that order.

The actual level of filler volume portion in nanocomposites can be suggested by total contents of interphase and filler as:

3 $$\varphi_{eff} = \varphi_{f} + \varphi_{i}$$

which results in the following form assuming Eq. (2) as:

4 $$\varphi_{eff} = \varphi_{f} \left( {1 + \frac{{2t_{i} }}{t}} \right)$$

Also, the percolating of 3D randomly organized graphite sheets in nanocomposites was expressed^60^ as:

5 $$\varphi_{p} = \frac{{27\pi D^{2} t}}{{4(D + d)^{3} }}$$

where “D” is the diameter of sheets and “d” is tunneling length. Since “D” (more than 1 μm) is much more than “d” (several nanometers), the latter equation can be simplified as:

6 $$\varphi_{p} = \frac{27\pi t}{{4D}}$$

However, Eq. (6) cannot consider the tunnels and interphase in the percolation beginning. The interphase regions form around the two-sides of nano-sheets. In addition, the tunneling spaces exist between two adjacent nano-sheets.

The effects of tunneling and interphase regions can be expected in the latter equation as:

7 $$\varphi_{p} = \frac{27\pi t}{{4D + 2(Dt + Dd)}}$$

When “$\varphi_{eff}$” and “$\varphi_{p}$” are replaced from Eqs. (4) and (7) into Eq. (1), the developed form of power-law model is expressed as:

8 $$\sigma = \sigma_{f} \left[ {\varphi_{f} + \frac{{2\varphi_{f} t_{i} }}{t} - \frac{27\pi t}{{2D(2 + t + d)}}} \right]^{b}$$

which correlates the conductivity to graphene dimensions, interphase depth and tunneling length. Generally, “b” determines the significances of graphene, interphase and tunneling properties on the conductivity of nanocomposites. The effect of “b” on the conductivity of samples and its optimized level will be given in the following sections.

## Results and discussion

### Comparisons of original and developed models to experimental data

The developed equations for percolation beginning and conductivity are valued by the experimental measurements from previous articles. Table 1 shows some reported samples and their graphene dimensions and processing methods. More details for materials and experimental setup were mentioned in the original references. Also, the percolation beginning of samples from the measurements of conductivity is reported. By associating Eq. (7) with the measured percolation beginning, the average values of interphase depth (t_i_) and tunneling length (d) are calculated. The different levels of these parameters are also represented in Table 1. The values of “t_i_” and “d” reveal that the interphase regions and tunneling effect play main roles in the percolation beginning and it is not possible to predict the percolation level in absence of these parameters. In other words, disregarding of interphase area and tunneling effect overpredicts the percolation beginning. The level of “t_i_” links to the interfacial interactions^61^. The polystyrene (PS)/graphene sample containing the thickest interphase (t_i_ = 8 nm) shows the strongest interfacial interactions among the reported samples. On the other hand, samples with t_i_ = 2 nm show the thinnest interphase as the poorest interfacial adhesion between polymer and graphene.

**Table 1:** The filler dimensions and percolation beginning of examples from original references and the calculations of tunneling length, interphase depth and “b” exponent by the mentioned equations.

Samples [Ref.]	Processing technique	t (nm)	D (μm)	$\varphi_{p}$	t_i_ (nm)	d (nm)	b
PVDF^1^/graphene^36^	Solution mixing	1	2	0.0030	2	3	7.0
PS^2^/graphene^62^	Solution mixing	1	2	0.0010	8	8	5.6
PET^3^/graphene^34^	Melt mixing	2	2	0.0050	3	4	4.6
Epoxy/graphene^63^	Solution mixing	2	2	0.0050	2	4	7.6

1: poly (vinylidene fluoride); 2: polystyrene; 3: poly (ethylene terephthalate).

**Figure 2:**
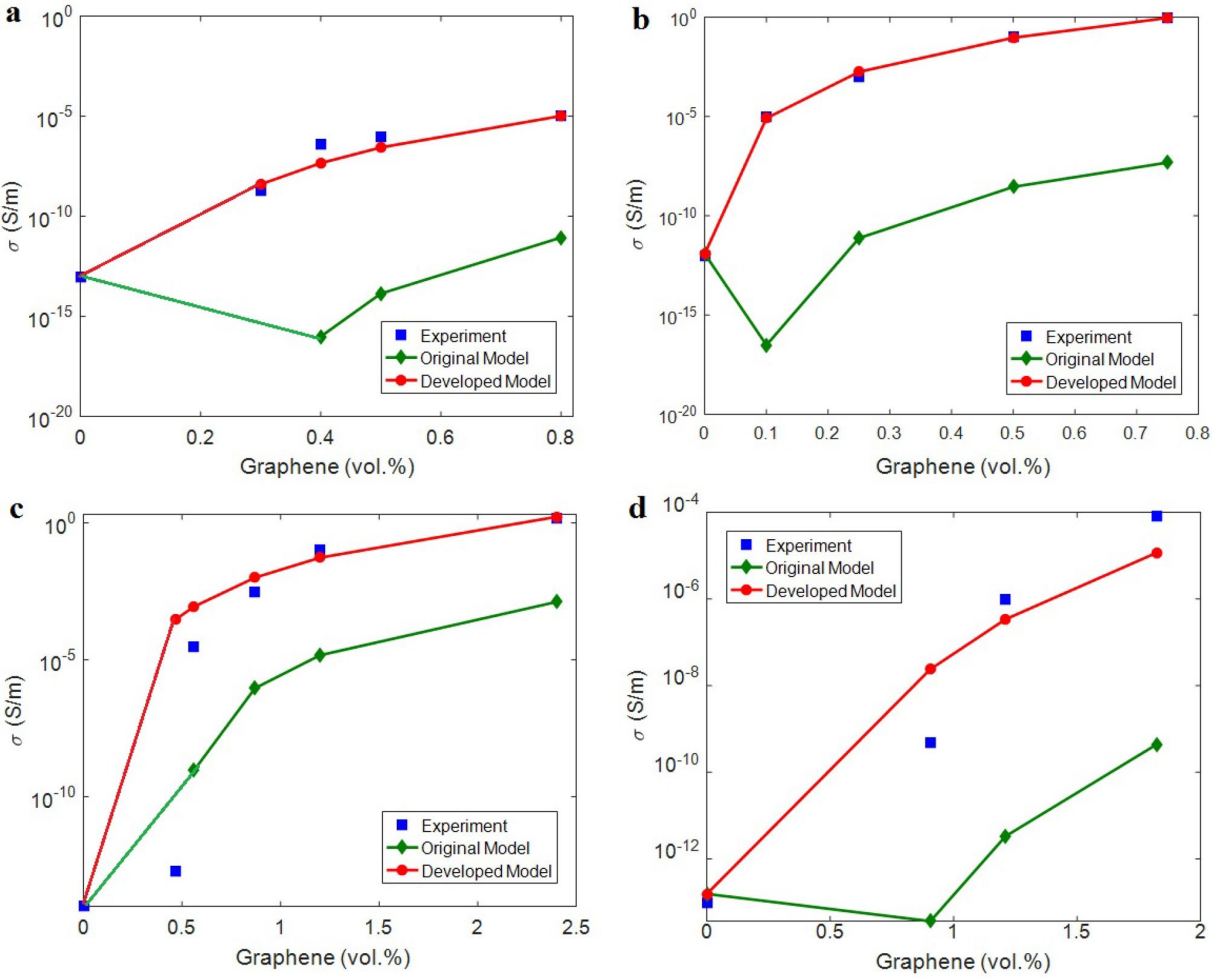
The experimented and theoretical values of conductivity by Eqs. (1) and (8) for (**a**) PVDF^36^, (**b**) PS ^62^, (**c**) PET^34^ and (**d**) epoxy^63^ graphene cases.

The average tunneling length “d” also varies from 3 to 8 nm in the examples. The maximum tunneling length, which can transfer the electrons was reported as 10 nm^64^. It can be suggested that the calculated “d” for the examples changes in a sensible range to provide the tunneling effect. The developed model in Eq. (8) is applied to foresee the conductivity of samples. Figure 2 shows the tested data and the calculations by the original (Eq. 1) and progressive (Eq. 8) models for poly (vinylidene fluoride) (PVDF)/graphene^36^**,** PS/graphene^62^, poly (ethylene terephthalate) (PET)/graphene^34^ and epoxy/graphene^63^ nanocomposites. The calculations of developed model illustrate the respectable arrangements with the tested data, but the original model underestimates the conductivity. Accordingly, the developed model assuming tunneling length and interphase regions can predict the conductivity of graphene system, while the original model is inappropriate.

The values of “b” exponent (Table 1) change from 4.6 to 7.6 for the present cases. The previous researchers have indicated that “b” depends on the dimensionality of filler nets in composites^58^, but it can be suggested that “b” relates to interphase depth and tunneling length in graphene products. Further studies in this area are necessary to show the dependency of “b” exponent to the actual parameters in polymer nanocomposites.

### Parametric studies

The impacts of model’s variables on the percolation beginning and conductivity are studied by the advanced equations. The parametric studies give the calculated results from the developed model. Actually, the parametric examinations confirm the roles of all parameters in the conductivity of nanocomposites using the developed model. Certainly, obtaining the calculated levels by experiment needs much time and cost, but 3D and contour plots only demonstrate and validate the reasonable effects of all parameters on the conductivity.

**Figure 3:**
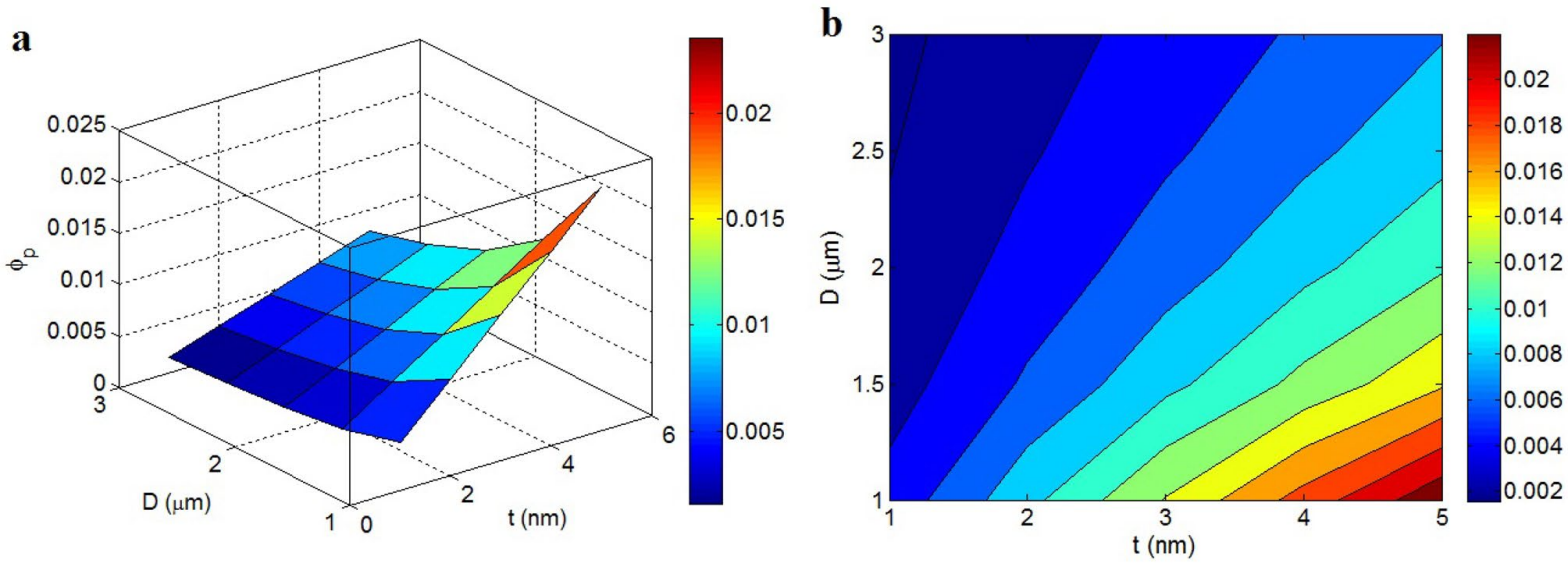
The percolation beginning by “t” and “D” (Eq. 7) at t_i_ = 2 nm and d = 5 nm: (**a**) 3D and (**b**) 2D patterns.

Figure 3 exhibits the stimuli of “t” and “D” on the percolation beginning (Eq. 7) at t_i_ = 2 nm and d = 5 nm. The maximum percolation beginning as 0.022 is calculated by t = 5 nm and D = 1 μm, while t = 1 nm and D = 3 μm produce the smallest percolation level. Actually, small “t” and high “D” yield the desirable level for percolation beginning in polymer graphene nanocomposites, while high “t” and low “D” negatively increase the percolation point. Accordingly, the percolation of thin and large graphene nano-sheets more quickly occurs in nanocomposites compared to thick and small ones.

The skinny and large nano-sheets harvest a big aspect ratio and big surface zone, which decrease the spaces between nano-sheets and grow the possibility of percolating and networking. Conversely, dense and small nano-sheets cannot introduce numerous inter-particle contacts, which weaken their percolating efficiency in nanocomposites. As a result, thin and large nano-sheets provide desirable percolation beginning by little amount of graphene. The previous articles reported an opposite correlation among percolation level and aspect ratio^63,65^. They indicated that a low percolation beginning is gotten by a big aspect ratio of graphene, i.e. thin and large nano-sheets. So, their predictions for percolation beginning agree with the developed equation in this study. In fact, the thin and big nano-sheets can occupy large regions in the nanocomposite, which facilitates the formation of nets by low filler concentration.

**Figure 4:**
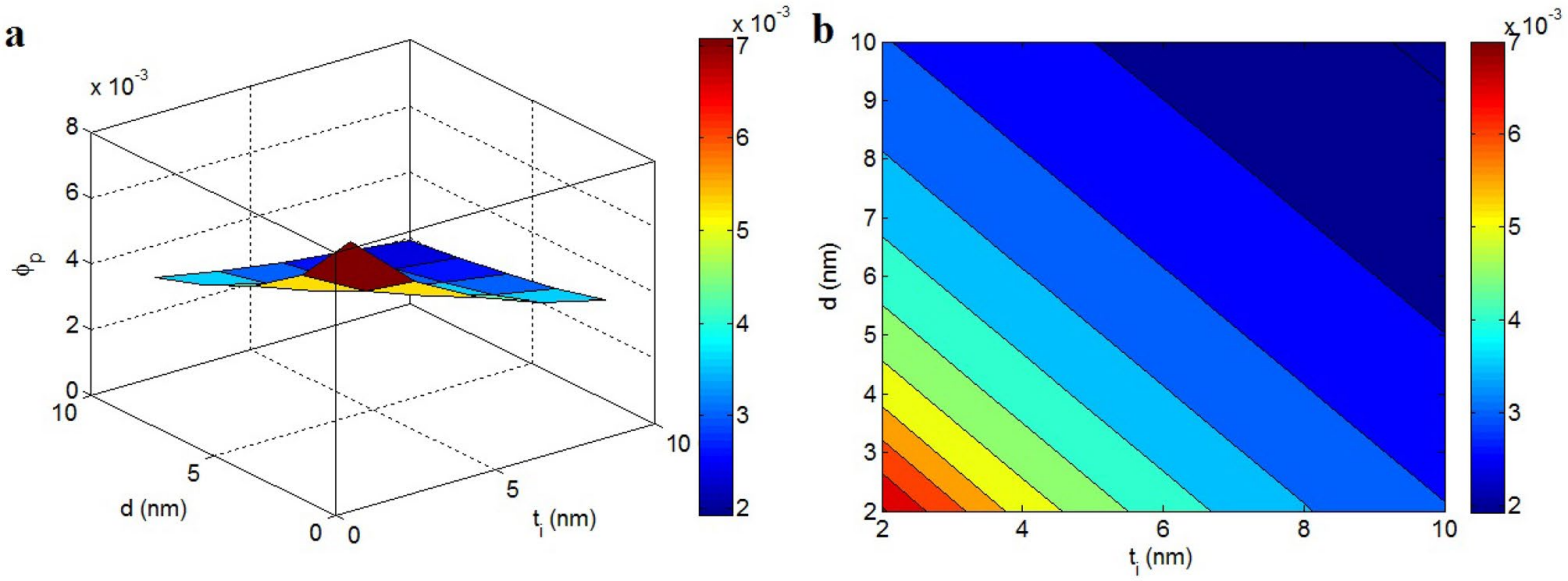
Effects of “t_i_” and “d” on percolation beginning at t = 2 nm and D = 2 μm: (**a**) 3D and (**b**) 2D intrigues.

The variations of percolation beginning at different series of “t_i_” and “d” are also illustrated in Fig. 4. The most desirable value of percolation beginning as 0.002 is acquired by t_i_ = 10 nm and d = 10 nm, although t_i_ = 2 nm and d = 2 nm raise the percolation level to 0.007. Accordingly, the highest values of “t_i_” and “d” can create the lowest percolation beginning in the samples. Instead, a high amount of nanoparticles is compulsory to get the percolation beginning in the case of thin interphase and short tunneling length.

The interphase regions around nano-sheets can generate the nets before the physical bonding of nano-sheets. So, a thick interphase about the large nano-sheets can cause the percolation beginning at very low volume portion of nanoparticles. Accordingly, the huge interphase regions in nanocomposites positively affect the percolating and networking of nanoparticles, because they form a high portion of polymer nanocomposites. However, the positive effect of interphase regions depends on their thickness and particle size according to Eq. (4). Undoubtedly, thin nano-sheets and thick interphase produce big interphase areas in nanocomposites, which advantageously affect the percolating efficiency of nanoparticles. The influences of interphase regions on the percolation beginning and toughness of CNT system were studied in the foregoing papers^49,66,67^, but the interphase stimuli on the percolation beginning and conductivity of graphene- filled system are studied in this paper for the first time.

The positive role of a large tunneling length in the percolation beginning of graphene is also reasonable. The graphene can form the nets when a short distance between adjacent nano-sheets exists as tunneling length. Actually, the neighboring nano-sheets separated by tunneling length can create the nets in the samples. Consequently, a large tunneling length can create the nets by few numbers of nano-sheets demonstrating the fast percolation by low filler concentrations. In conclusion, the developed equation suitably shows the powers of interphase depth and tunneling length on the percolation beginning.

**Figure 5:**
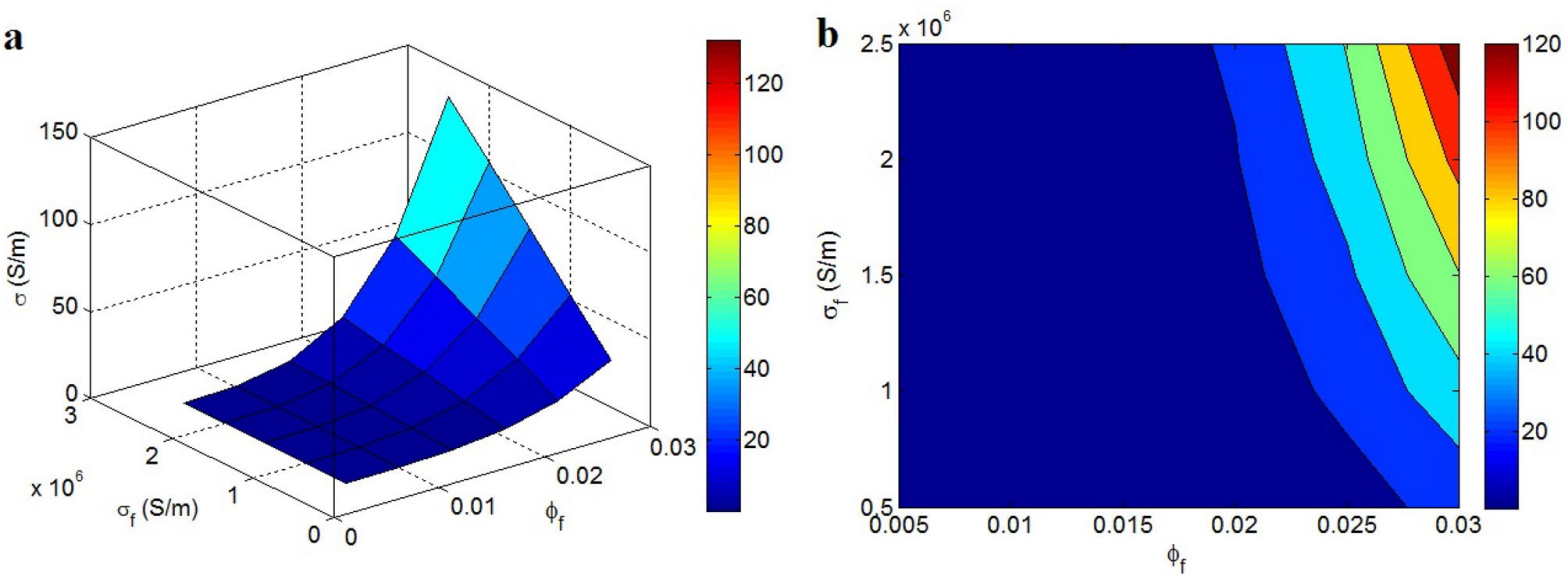
(**a**) 3D and (**b**) 2D schemes for the impacts of “$\varphi_{f}$” and “σ_f_” on the conductivity (D = 2 μm, t = 2 nm, t_i_ = 2 nm, d = 5 nm and b = 4).

Figure 5 shows the conductivity at unlike concentration and conduction of graphene according to Eq. (8) at D = 2 μm, t = 2 nm, t_i_ = 2 nm, d = 5 nm and b = 4. Obviously, these parameters directly affect the conductivity, because the graphene nanofiller is more conductive than polymer matrices. When $\varphi_{f}$ = 0.03 and σ_f_ = 2.5*10^6^ S/m, the conductivity of 120 S/m is attained for nanocomposite, but the nanocomposite remains approximately insulated when $\varphi_{f}$ < 0.019. The concentration and conduction of graphene play important roles in the conductivity. A little content of graphene may not touch the percolation beginning or harvest poor nets, which insignificantly improve the conductivity. Additionally, the polymer matrices are commonly insulated and thus, the conduction of graphene nanoparticles controls the conductivity. The extraordinary conduction of graphene evidently produces a conductive nanocomposite.

**Figure 6:**
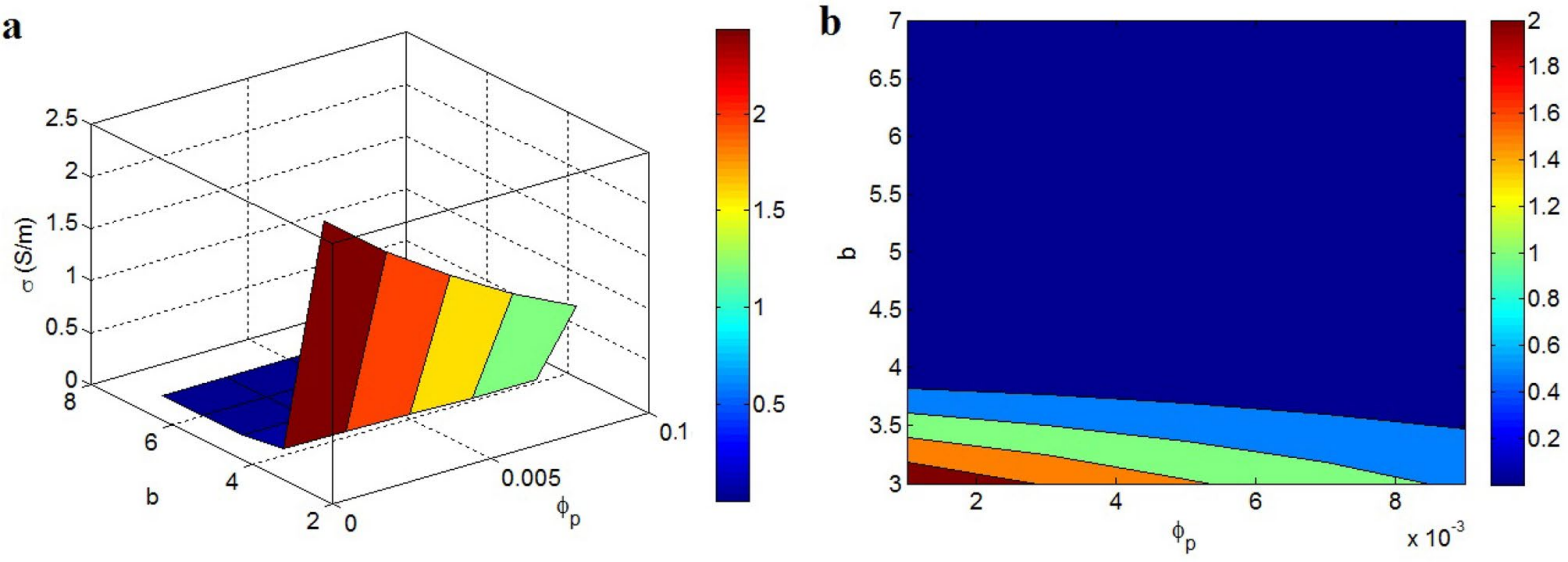
The stimuli of “$\varphi_{p}$” and “b” on the conductivity: (**a**) 3D and (**b**) 2D diagrams.

Figure 6 also establishes the connection of conductivity to “$\varphi_{p}$” and “b” at $\varphi_{f}$** = **0.01 and σ_f_ = 10^5^ S/m. The maximum conductivity as 2 S/m is obtained by the slightest amounts of “$\varphi_{p}$” and “b”, i.e. $\varphi_{p}$ = 0.001 and b = 3, while the conductivity approaches to 0 when b > 3.8. Accordingly, small percolation beginning and low “b” exponent produce high conductivity; nevertheless a deprived conductivity is detected at great “b”. In other words, only smaller values of both percolation beginning and “b” cause better conductivity.

A poor percolation beginning produces the conductive nets of graphene nano-sheets at low filler contents. So, high conductivity is shown by low filler concentrations in this condition and the addition of nano-sheets above percolation beginning increases the net density and dimensions. Obviously, the dense and big nets can enlarge the transferring of electron in the nanocomposites. However, a high percolation beginning shows the networking of graphene nano-sheets at high filler concentration. In this state, the nanocomposite can show the conductivity only by great filler concentration. In addition, a high value of “b” considerably decreases the conductivity, but the exact definition for this parameter is not available. It seems that the “b” links to filler size, net dimensionality, interphase scale and tunneling length. It can be thought that the big values of “b” demonstrate the inappropriate conditions in nanocomposite for conductivity.

**Figure 7:**
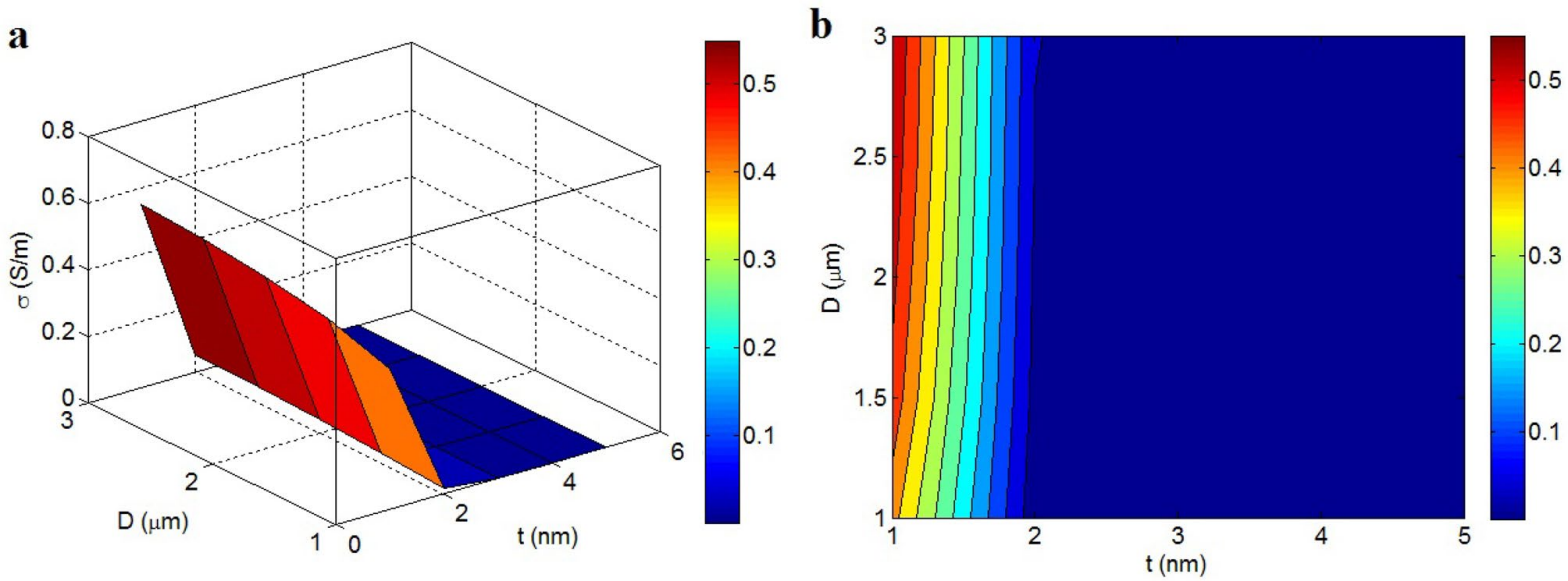
Connections of conductivity to “t” and “D” by (**a**) 3D and (**b**) 2D pictures.

The conductivity linking to “t” and “D” are designed in Fig. 7 at t_i_ = 2 nm, $\varphi_{f}$ = 0.01, σ_f_ = 10^5^ S/m, d = 5 nm and b = 4. The uppermost conductivity is witnessed by the thinnest and the biggest nano-sheets. The supreme conductivity of 0.55 S/m is attained at t = 1 nm and D = 3 μm. However, thick graphene nano-sheets (t > 2 nm) significantly weaken the conductivity. As a result, the dimensions of graphene nano-sheets control the conductivity. It can be suggested that the poor dispersion of nanoparticles thickening the graphene nano-sheets negatively affects the conductivity.

The thick and small nano-sheets show small surface area, which decreases the contacts between nanoparticles in nanocomposite. So, the percolating and networking of thick and small nano-sheets at low concentrations is difficult, which results in poor conductivity. Also, the thick and small nano-sheets produce small and light nets after percolation beginning, which are not efficient for electron transportation and conductivity. Therefore, thick and short nano-sheets cannot produce proper conditions for percolating of nanoparticles and achieving a desirable conductivity. In summary, the undesirable characters of thick and small graphene nano-sheets in the conductivity can be interpreted by their contribution to the aspect ratio and surface area of conducting filler. Hence, the established model accurately exhibits the stimuli of these factors on the conductivity.

**Figure 8:**
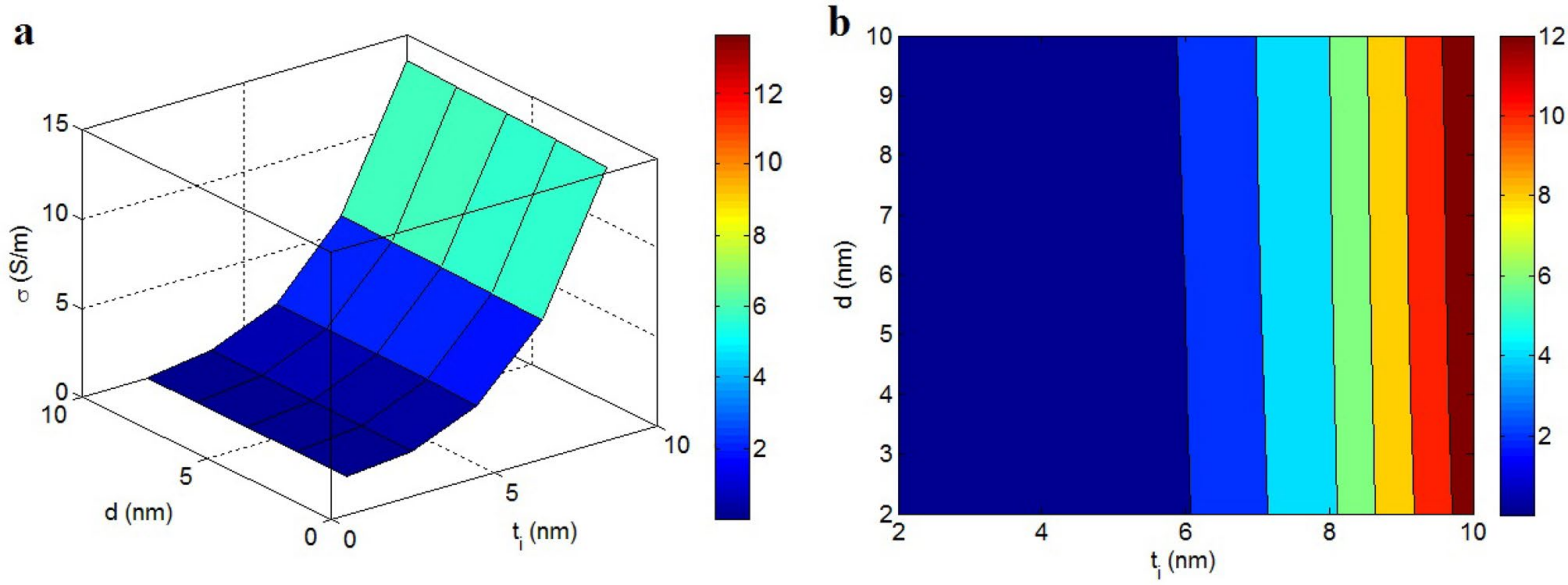
(**a**) 3D and (**b**) 2D designs to express the conductivity at various levels of “t_i_” and “d”.

Figure 8 also reveals the roles of “t_i_” and “d” as interphase deepness and tunneling size in the conductivity based on Eq. (8) at σ_f_ = 10^5^ S/m, t = 2 nm, D = 2 μm, $\varphi_{f}$ = 0.01 and b = 4. The effect of interphase depth on the conductivity is more emphasized compared to tunneling length, because the interphase depth significantly influences the actual filler portion (Eq. 4). As observed, the interphase depth less than 6 nm cannot improve the conductivity, while the thickest interphase (t_i_ = 10 nm) produces the best conductivity of 12 S/m. Therefore, the conductivity directly changes by interphase depth, but the role of tunneling length is negligible.

The significant aspect ratio of graphene nano-sheets intensifies the effect of interphase on the common features of system, because the interphase regions can occupy a high part of nanocomposite. The thick interphase areas harvest the nets at lower filler portions, because they form the net structures before the real attachment of fillers. So, thick interphase regions attach to the nets and improve the net properties and conductivity.

The contributions of interphase regions to the real filler portion and percolation beginning are assumed by Eqs. (4) and (7), respectively. A profuse interphase confidently affects both percolation beginning and actual filler portion, but the tunneling length only changes the percolation beginning and does not play a role in the efficient filler concentration. Thus, the interphase depth role in the conductivity is more important than that of tunneling length. However, the researches on the conductivity have reported the negative impact of tunneling length on the conductivity^68,69^.

Parametric examinations validated the developed model by a simple methodology using 3D and contour plots. The effects of all parameters on the conductivity were revealed by the plots, but the analysis of correlation among the parameters was not given, because the proper roles of all factors in the conductivity of samples are sufficient for the validation of developed model. Actually, 3D and contour plots demonstrate the roles of parameters in the conductivity and the trends are not different by dissimilar couples of parameters.

## Conclusions

The power-law model was progressed for graphene-filled composites by the impacts of tunneling length, interphase deepness and filler size on the actual concentration and percolation beginning of graphene. The developed equations were examined by the experimental measurements and the influences of several factors on the percolation beginning and conductivity were discussed. The experimental levels of percolation beginning show the development of dense interphase and long tunnels in the examples and thus, the nonattendance of these factors overestimates the percolation beginning. Also, the novel model can successfully foretell the conductivity for the examples. A poor percolation beginning is gotten by thin and large graphene nano-sheets as well as by thick interphase and long tunneling length. However, filler dimensions more significantly change the percolation beginning compared to other parameters. In addition, the high values of filler concentration, filler conduction, filler diameter and interphase depth cause good conductivity, whereas a higher conductivity is attained by lower percolation beginning, minor “b” exponent and thinner nano-sheets. Also, the tunneling length shows negligible influence on the nanocomposite conductivity. The most influences on the conductivity are introduced by the conduction and concentration of graphene and interphase depth.

## Data Availability

The datasets used and/or analyzed during the current study available from the corresponding author on reasonable request.
